# Predictors of glucose control in children and adolescents with type 1 diabetes: results of a cross-sectional study in Cameroon

**DOI:** 10.1186/s13104-017-2534-8

**Published:** 2017-06-12

**Authors:** Loveline L. Niba, Benedikt Aulinger, Wilfred F. Mbacham, Klaus G. Parhofer

**Affiliations:** 1 CIHLMU Center for International Health at Ludwig-Maximilians-Universität, Munich, Germany; 2 Department of Biochemistry, Catholic University of Cameroon (CATUC), P.O. Box 782, Bamenda, Cameroon; 3 Department of Medicine II-Grosshadern, Ludwig-Maximilians-Universität, Marchioninistr. 15, 81377 Munich, Germany; 4 Department of Physiology and Biochemistry, Faculty of Medicine, University of Yaoundé I, P.O. Box 8094, Yaoundé, Cameroon

**Keywords:** Predictors, Outcome, HbA1c, Type 1 diabetes, Children/adolescents

## Abstract

**Background:**

In sub-Saharan Africa the prognosis of children with type 1 diabetes is poor. Many are not diagnosed and those diagnosed have a dramatically reduced life expectancy (less than one year). The purpose of this study was to identify the predictors of glucose control in children and adolescents with type 1 diabetes.

**Methods:**

This hospital based cross-sectional study involved 76 children/adolescents (35 boys and 41 girls, mean age of 15.1 ± 3.1 years) with type 1 diabetes included in the “Changing Diabetes in Children” (CDiC) program and attending the clinics for children living with type 1 diabetes in the North West Region of Cameroon. Data on glycosylated haemoglobin (HbA1c) was obtained from hospital records of participants. Information on socio-demographic characteristics and diabetes related practices were obtained from participants using a structured questionnaire. Odds ratios (OR) were calculated using logistic regression models to assess the association between determinants and good glyceamic control.

**Results:**

The study population had a mean HbA1c of 10.3 ± 2.9%. Bivariate analysis indicated that having a mother as the primary caregiver (OR 0.07, 95% CI 0.02–0.2), being on 2 daily insulin injections (OR 0.2, 95% CI 0.1–0.5) and good blood glucose monitoring (BGM) adherence (OR 0.1, 95% CI 0.04–0.3) were significantly (*p* < 0.001) associated with better HbA1c. Minimal/moderate caregiver involvement in BGM (OR 7.7, 95% CI 2.7–22.0) and insulin injection (OR 14.9, 95% CI 4.8–46.5) were significantly (*p* < 0.001) associated with poor outcome. Multivariate analysis showed that having a mother as the primary caregiver (OR 0.02, 95% CI 0.002–0.189) was an independent predictor of good glucose control.

**Conclusions:**

This study has shown that the mother’s involvement in the diabetes management of their children and minimal/moderate caregiver involvement in the task of insulin injection are the most important determinants for good and poor glucose control respectively. It is currently unclear whether the direct involvement of the mother is causal or whether “mother as a primary caregiver” is just an indicator for a setting in which good diabetes treatment is possible.

**Electronic supplementary material:**

The online version of this article (doi:10.1186/s13104-017-2534-8) contains supplementary material, which is available to authorized users.

## Background

In sub-Saharan Africa the prognosis of children with type 1 diabetes is poor. Many are not diagnosed and those diagnosed have a dramatically reduced life expectancy (less than one year) [1]. Diabetes management requires continuous medical care and patient self-management education to prevent acute complications and the risk of long-term complications [2]. However, pediatric diabetes management has remained a major challenge to the patient, the healthcare provider as well as family members of the patients [3, 4, 5].

Despite tremendous evidence that maintaining strict glycaemic control in type 1 diabetics prevents long-term and short-term adverse health outcomes [6, 7], many patients still remain poorly controlled [8, 9], even in clinical trial settings [10]. Factors associated with good or poor glycaemic control differ between adults and children.

In children, demographic characteristics such as age [5, 11], gender [12], socioeconomic status [13, 14] and family living arrangements [15, 16] are thought to be predictive of a patient`s glycosylated haemoglobin (HbA1c) level. In addition, diabetes-related characteristics including; frequency of self-monitoring of blood glucose [4, 17, 18], diet [19], diabetes duration [20, 21], regularity of clinic visits [22, 23, 24], insulin regimen [6] and family involvement in diabetes related tasks [25] are also associated with the level of glycaemic control of patients.

However, these factors have been identified in developed countries and it is unclear whether the same or other factors determine glucose control in settings with very limited health resources. This is particularly important as glycaemic control in children with type 1 diabetes seems to improve in industrialized countries while it is not improving in Sub-Saharan Africa [27, 28].

The aim of this study is to identify factors that predict the outcome of children with type 1 diabetes in the North West Region of Cameroon.

## Methods

### Subjects

The data used in this study was obtained from a hospital based cross-sectional study of data collected between January and August 2014, involving children and adolescents aged 0–18 years attending the outpatient clinics for children living with diabetes in 2 hospitals in the North West Region of Cameroon (Bamenda Regional hospital—Bamenda and the Banso Baptist hospital—Kumbo). These clinics run once every week. A total of 76 children (35 boys and 41 girls) were studied.

These children are reviewed at least once every three months by the diabetic nurse in charge of the clinic who also communicates with the physician in charge of the children regarding clinical care issues. During each clinic visit, height, weight, systolic and diastolic blood pressure, foot examinations and glucometer readings are reviewed.

In addition, all patients attending these clinics are provided with insulin at no cost through the International Diabetes Federation (IDF), “Changing Diabetes in Children” (CDiC) program. Also, they are provided with blood glucose monitors, glucose strips, and diaries for measuring and recording of their blood glucose at home. These children/adolescents are encouraged to monitor their glucose level at least 3 or 4 times a day and record the information in their diaries. The blood glucose levels are then used by the clinicians to alter appropriately the insulin regimen. The patients are either on a 2 daily insulin injection regimen or on a multiple daily insulin injection regimen.

A written consent that explained the purpose of the study was distributed to parents/guardians of the study participants and the heads of the clinics. Also, the research staff had to explain the purpose of the study to the participants before data collection. Those who consented to the study were asked to sign the consent form and assent was obtained from children above 10 years of age.

### Study procedure and parameters

During a regularly scheduled clinic visit, the research staff (assisted by a nurse in each clinic) met with each child/adolescent and parent or caregiver and jointly completed a structured questionnaire (which was piloted before the study) to gather information on socio-demographic and diabetes related practices. The questionnaire was straightforward and we needed the dyad in the completion of the questionnaire to ensure the information provided was correct. For children below the age of 10, parents were interviewed and were assisted to complete the questionnaire. The same applied for adolescents who could not read or write, the questionnaire was also completed for them after interviewing them and the responses were confirmed from parents/caregivers who accompanied these children. However, adolescents who were literate and were not accompanied by any parent or caregiver were asked to complete the questionnaire in the presence of the research staff assisted by a nurse (Additional file 1).

Socio-demographic characteristics included: age, gender, family living arrangements, family history of diabetes, degree of urbanization, socioeconomic status and primary caregiver.

In a household the primary caregiver was defined as the person in the family most involved in the care of the diabetic child.

Diabetes-related characteristics included: age at onset of type 1 diabetes, diabetes duration, insulin regimen, insulin adherence, dietary adherence and blood glucose monitoring (BGM) adherence, regularity of clinic visits and family involvement in diabetes related tasks (insulin injection and blood glucose monitoring).

Diabetes-related practices were self-reported by parents and/or children on the questionnaires.

Patient adherence to insulin was determined by the number of insulin doses missed in the last one week and it was graded as good—for those who never missed any dose, average—for those who missed 1–3 doses in the last one week and poor—for those who missed more than 3 doses in the last one week. In addition, reasons for missing the insulin doses were recorded.

Adherence to diet was assessed using a 24-h dietary recall and was graded using a score derived from dietary guidelines given at the clinic based on meal frequency and meal content. In the scoring process of meal content, each time a contra-indicated food (such as soft drinks, added sugar, animal fat) was consumed it was given a score of—1. A maximum score of 8 was obtained for dietary adherence; a score of less than 4, between 4 and 6, and greater than 6 were interpreted as poor, average and good respectively.

Blood glucose monitoring (BGM) adherence was graded as good—for those who measured their blood glucose 3 or more times a day, average—for those who measured their blood glucose 1–2 times a day, and poor—for those who measured their blood glucose less than once a day.

Family involvement in diabetes related tasks was assessed by the degree of involvement of parents/caregivers in insulin administration and BGM. This was then graded as minimal, moderate and maximal involvement using a modified scale used in the study by Anderson et al. [25].

Caregiver involvement in insulin injections was determined by the number of doses of insulin injections injected or supervised by the caregiver (which was age-adjusted) in the last 24 h and it was graded as minimal—no caregiver participation, moderate—caregiver injected/supervised only half of the injections and optimal- caregiver gave all the injections.

Also, caregiver involvement in BGM was determined by the degree of participation of the caregiver in the task of BGM (which was age-adjusted) was graded as minimal—no caregiver participation, moderate–caregiver reminded the child to check blood glucose, asked the child about the blood glucose level or entered the glucose level in the diary and maximal-caregiver sets up the meter or did the finger prick and registered the results in the diary.

Family living arrangement was categorized as follows; living with both parents, living with a single parent, sibling, family relative or an orphan.

Degree of urbanization was defined by the area of resident of the patient as follows; urban and rural.

Regularity of clinic visits was determined by the number of times the patient attended the outpatient diabetic clinic in the last 6 months before the study given the fact that cost of transportation to the clinic was covered by the program.

Data on date of birth, height, weight, gender as well as clinical/biochemical parameters (HbA1c, insulin requirements, fasting blood glucose, postprandial glucose, systolic and diastolic blood pressure) both at diagnosis and during the study period which are part of the routine care in the clinics were collected from the hospital records of each patient.

### Anthropometric measurements

Height and body weight were measured by the clinic nurses ensuring that standard protocols were respected. Height was measured to the nearest 0.1 cm using a portable stadiometer (Seca 213, Germany). The body weight of each patient was measured using a digital scale (Omron BF 511, Japan) to the nearest 0.1 kg in order to accurately determine the insulin dose per kilogram body weight. The body mass index (BMI) of each participant was then calculated [29].

### Glycaemic control

HbA1c which was the outcome of interest was measured using a BIO-RAD in2it™ Analyzer (UK) which makes use of a BIO-RAD A1c system test cartridge at the time of the index visit. Blood glucose measurements (fasting blood glucose and postprandial glucose) were measured using an Accu-Chek Active blood glucose monitoring system (Germany).

### Socioeconomic status (SES)

This was assessed using the Cameroon public service system of occupation classification and the civil servant categories A, B, and C were used to categorize patients into high, middle and low SES respectively [30]. Individuals not working in the public sector were also assigned to these categories based on their income or profession. This information was provided by the parents/caregivers of the patients. Each child was assigned to a socioeconomic status category based on the highest level of SES of either parent. Furthermore, parental level of education was also assessed as a measure of SES using the questionnaire and four categories were established: no formal education (no elementary education), primary (1–6 years of education), secondary (7–13 years of education) and higher education (greater than 13 years of education).

### Ethical and administrative clearances

Ethical approval was obtained from the National Ethics Committee (NEC) of the Ministry of Public Health, Cameroon. Administrative clearance was obtained from the Regional Delegation for Public Health of the North West Region of Cameroon. Hospital clearances were also obtained from the Bamenda Regional Hospital and Banso Baptist Hospital. All the children and adolescents gave written informed consent before any data collection was done. Also, parental informed consent was obtained for children less than 16 years of age after distributing a written consent that explained the purpose of the study to the parents/guardians of the study participants.

### Statistical analysis

SPSS for windows version 20 was used for data analyses. Continuous variables were tested for normality using the Kolmogorov–Smirnov (K–S) test. The anthropometric variables (height, weight and BMI) were standardized for age and gender (Z scores) using the WHO AnthroPlus software. This package uses the WHO 2007 growth reference data [31]. Patients’ sociodemographic characteristics and diabetes specific variables were summarized using frequency distribution tables. The mean HbA1c was compared across diabetes specific characteristics and different treatment regimens using a parametric *t* test. In addition, a paired *t*-test was used to compare the differences in means of clinical and biochemical parameters of patients at diagnosis and during the study period. Unequally distributed variables were analyzed using the non-parametric Mann–Whitney *U* test. The association between caregiver involvement in diabetes related tasks and patient adherence was tested using Chi square test. The study population was then divided into two groups of glycaemic control as measured by HbA1c (poor glycaemic control, HbA1c >9.0% and good glycaemic control, HbA1c ≤9%) [10]. In addition, the frequencies of poor glycaemic control by potential determinants were calculated and this was followed by calculation of odds ratios (OR) using a univariate binary logistic regression analysis. Further, a multivariate binary logistic regression analysis (adjusting for age and gender) using a stepwise forward technique was performed to determine the independent predictors of glycaemic control using all the variables that were significant in the univariate analysis. A *p* value of 0.05 was used to indicate statistical significance.

## Results

This study included 76 children/adolescents with type 1 diabetes mellitus and 74% had been living with diabetes for more than 2 years. Table 1 shows the main characteristics of the study population. More than 50% of the study participants were female. The mean age at diagnosis was 15.1 (95% CI 14.4–15.8) years with girls having a slightly higher mean age at diagnosis compared to boys (15.4 ± 2.6 vs 14.8 ± 3.7 years). The mean duration of diabetes for the study population was 3.8 (95% CI 3.1–4.5) years. A majority of the study participants were living with both biological parents, had a mother as the primary caregiver and received three or more insulin injections daily. In addition, more than 50% of the patients were of low/middle SES.

**Table 1:** Descriptive characteristics of the study population, N [% (95% CI)], N = 76

Variables	N	Frequency		Mean	(95% CI)
		%	(95% CI)		
Age				15.1	(14.4–15.8)
First tertile (4–14 years)	25	32.9	(23.4–44.1)		
Second tertile (15–16 years)	25	32.9	(23.4–44.1)		
Third tertile (>16 years)	26	34.2	(24.5–45.4)		
Gender					
Male	35	46.1	(35.3–57.2)		
Female	41	53.9	(42.8–64.7)		
Body mass index (BMI) (kg/m^2^)				23.3	(22.1–24.5)
Underweight (<18.5)	6	7.9	(3.7–16.2)		
Normal (18.5–25.0)	52	68.4	(53.7–77.8)		
Overweight + obese (>25)	18	23.7	(15.5–34.4)		
Family structure					
Both parents living together	46	60.5	(49.3–70.8)		
Single parent	17	22.4	(14.5–32.9)		
Not living with parents	8	10.5	(5.4–19.4)		
Orphan	5	6.6	(2.8–14.5)		
Primary caregiver					
Mother	45	59.2	(48.0–69.6)		
Father	10	13.2	(7.3–22.6)		
Sibling	9	11.8	(6.4–21.0)		
Other	12	15.8	(9.3–25.6)		
Duration of diabetes (years)				3.8	(3.1–4.5)
<2	20	26.3	(17.7–37.2)		
2–5	37	48.7	(37.8–59.7)		
>5	19	25.0	(23.4–44.1)		
Insulin regimen					
2 daily injection	31	40.8	(30.4–52.0)		
Multiple daily injection	45	59.2	(48.0–69.6)		
Degree of urbanization					
Urban	30	39.5	(29.3–50.7)		
Rural	46	60.5	(49.3–70.8)		
Socioeconomic status					
Low	53	69.7	(58.7–78.9)		
Middle	9	11.8	(6.4–21.0)		
High	14	18.4	(11.3–28.6)		

*CI* confidence interval

The mean HbA1c of the study population was 10.3 ± 2.9% and only 24% of the children attained the HbA1c target level of <7.5%. However, there was a significant decrease in HbA1c from diagnosis (11.1%) to the study period (10.3%) (*p* = 0.011). Girls on average had a lower mean HbA1c (9.9%, 95% CI 9.0–10.8) compared to boys (10.8%, 95% CI 9.7–11.9). However, this was not statistically significant.

Table 2 shows the diabetes-related characteristics by glycaemic control of the patients. Glycaemic control (HbA1c) was more likely to be better among children having a mother as a primary caregiver (8.7%, 95% CI 8.0–9.4) compared to those having a father, a sibling or another family member as caregiver (12.7%, 95% CI 11.9–13.4). Also, children in the third age tertile had a lower mean HbA1c (9.8%, 95% CI, 9.1–10.5) compared to those in the first age tertile (10.8%, 95% CI, 10.2–11.4, *p* = 0.209). However, there was no significant linear trend for HbA1c to decrease with increasing age (*p* = 0.228). In addition, there was no significant difference in glycaemic control between the different categories of diabetes duration, family living arrangements and family history of diabetes (*p* > 0.05).

**Table 2:** HbA1c of patients by specific diabetes characteristics, [mean (95% CI)]

Variables	HbA1c		*p* value
	Mean	95% CI	
Age tertiles			
First (4–14 years)	10.8	(10.2–11.4)	
Second (15–16 years)	10.3	(9.6–11.0)	
Third (>16 years)	9.8	(9.1–10.5)	0.482^a^
Duration of diabetes (years)			
<2	10.5	(9.1–11.9)	
2–5	10.1	(9.1–11.1)	
>5	10.5	(9.2–11.8)	0.860^a^
Insulin Regimen			
2 daily injections	8.9	(7.9–9.9)	
Multiple daily injection	11.2	(10.4–12.0)	0.001*
Family structure			
Both parents living together	10.2	(9.3–11.1)	
Others	10.4	(9.3–11.5)	0.771*
Primary caregiver			
Mother	8.7	(8.0–9.4)	
Others	12.7	(11.9–13.4)	<0.001*
Clinic visits in the last 6 months			
1–3 times	11.5	(10.7–12.3)	
>3 times	8.7	(7.8–9.6)	<0.001*
Socioeconomic status			
Low/middle	10.9	(10.2–11.6)	
High	9.1	(8.5–9.7)	0.014*
Family history of diabetes			
Positive family history	10.5	(9.1–11.9)	
No family history	10.2	(9.4–11.0)	0.654*

*CI* confidence interval

* Calculated using students’ independent *t* test

^a^Calculated using one-way ANOVA

Bivariate analysis (unadjusted associations between poor glycaemic control and individual factors) in Table 3 indicated that having a mother as the primary caregiver (OR 0.07, 95% CI, 0.02–0.2) being on 2 daily insulin injections (OR 0.2, 95% CI, 0.1–0.5), good adherence to blood glucose monitoring (OR 0.1, 95% CI, 0.04–0.3) and good adherence to insulin injection (OR 0.3, 95% CI, 0.1–0.8) were significantly (*p* < 0.05) associated with good glucose control as indicated by HbA1c, while age (OR 1.1, 95% CI 0.4–3.2), diabetes duration (OR 0.9, 95% CI 0.3–2.9) and socioeconomic status (OR 2.4, 95% CI 0.9–6.5) did not show any significant association (*p* > 0.05) with glycaemic control.

**Table 3:** Frequency and odds ratio for the association between poor glycaemic control and determinants (bivariate analysis)

Determinants	N	Poor glycaemic control			*p* value
		Frequency	OR	95% CI	
Age tertiles					
Third (>16 years)	26	50.0	1.1	(0.4–3.2)	0.886
Second (15–16 years)	25	52.0	1.5	(0.5–4.5)	0.474
First (4–14 years)	25	60.0	ref		
Diabetes duration (years)					
>5	19	52.6	0.9	(0.3–2.9)	0.928
2–5	37	51.4	1.4	(0.4–4.8)	0. 643
<2	20	60.0	ref		
Primary caregiver					
Mothers	45	31.1	0.07	(0.02–0.2)	<0.001
Others	31	87.1	ref		
Insulin regimen					
2 daily injection	45	21.9	0.2	(0.1–0.5)	<0.001
Multiple daily injection	31	71.1	ref		
Insulin adherence					
Good	26	34.6	0.3	(0.1–0.8)	0.017
Poor/average	50	64.0	ref		
BGM adherence					
Good	43	32.6	0.1	(0.04–0.3)	<0.001
Poor/average	33	81.8	ref		
Dietary adherence					
Poor/average	69	52.2	2.3	(0.4–12.6)	0.341
Good	7	71.4	ref		
Caregiver involvement in insulin injection					
Minimal/moderate	37	83.8	14.9	(4.8–46.5)	<0.001
Maximal	39	25.6	ref		
Caregiver involvement in BGM					
Minimal/moderate	34	79.4	7.7	(2.7–22.0)	<0.001
Maximal	42	33.3	ref		
Clinic visits in the last 6 months					
1–3 times	44	70.5	5.2	(1.9–14.1)	0.001
>3 times	32	31.3	ref		
Socioeconomic status					
Low/middle	53	60.4	2.4	(0.9–6.5)	0.091
High	23	39.1	ref		
Degree of urbanization					
Rural	46	60.0	1.5	(0.6–3.8)	0.394
Urban	30	50.0	ref		

*OR* odds ratio (adjusted for age and gender), *CI* confidence interval, *BGM* blood glucose monitoring: Poor glycaemic control; HbA1c >9.0%

We observed that 63% of patients being treated on 2 daily insulin injections had good glycaemic control compared to 37% of those on multiple daily insulin injections. Also more than 80% of the study participants who checked their blood glucose 3 or more times a day had good glycaemic control compared to 17% of those who had 2 or less blood glucose checks a day. Further, 58% of children with poor glycaemic control had a positive family history of diabetes.

Insulin adherence was reported to be good in less than 40% of the study population while 22% reported poor adherence to insulin. More than 45% of the children and adolescents reported non-adherence to insulin to be due to forgetfulness, lack of food and inconvenience injecting insulin in school and other public. Also, more than 50% of the study participants reported good adherence to BGM. However, more than 35% of the study participants cited laziness as the main reason for non-adherence to BGM. Adherence to the dietary regimen prescribed at the clinic was average in a majority (82%) of the patients. In addition, there was a significant association between BGM adherence by age tertiles (Chi square value = 10.4, df = 4, *p* = 0.034).

In this study it was observed that caregiver involvement in the diabetes-related tasks of the child varied with the type of task. 88% of the children reported maximal parental involvement in the task of BGM compared to 58% in the task of insulin injection. Maximal caregiver involvement in the task of BGM was significantly associated with good patient adherence to BGM (Chi square = 44.5; df = 2; *p* < 0.001). However, no such relationship was observed in the task of insulin injection.

Multivariate analysis (Table 4), showed that having a mother as the primary caregiver (OR 0.02, 95% CI 0.002–0.189, *p* < 0.001) was significantly associated with good glucose control. Also, participants who had minimal/moderate caregiver involvement in the task of insulin injection had an increased risk of poor glucose control (OR 26.8, 95% CI 4.4–56.1, *p* < 0.001). This multivariate model shows a statistically independent association between having a mother as the primary caregiver and good glycaemic control.

**Table 4:** Multivariate binary logistic regression analysis with HbA1c (%) as dependent variable, (odds ratios adjusted for age and gender)

	B	Standard error	Odds ratio (OR)	(95% CI)	*p* value
Primary caregiver	−3.436	1.082			
Mother			0.02	(0.002–0.189)	0.001
Others				ref	
Caregiver involvement in insulin injection	3.617	1.046			
Minimal/moderate			26.8	(4.4–56.1)	0.001
Maximal				ref	
Constant	1.795	1.557			

*OR* odds ratio, *CI* confidence interval

Although not part of this cross-sectional study we observed that 3 of the 76 children died during the observation period (2 children from presumed hypoglycemia and 1 from presumed diabetic ketoacidosis).

## Discussion

Achieving good glycaemic control is the cornerstone in the management of type 1 diabetes as it is essential for the prevention of short and long-term complications. This study sets out to identify the factors that predict good glucose control in children with type 1 diabetes in Cameroon. This study has demonstrated that having a mother as a primary caregiver is an important predictor of good glycaemic control among children with type 1 diabetes. Also, it was shown from the multivariate analysis that children with minimal/moderate caregiver involvement in the task of insulin injection were at risk of poor glucose control as measured by HbA1c.

We found that the mean HbA1c of the study population was 10.3 ± 2.9% and that more than three-quarters (76%) of the patients in this study were not adequately controlled (HbA1c >7.5%), values similar to those obtained in Tanzania and Kenya [27, 32] but worse than values observed in developed countries [14, 33]. A number of factors including irregular supply of insulin, non-availability of structured diabetes programs, and lack of acceptance of chronic diseases within the society (employer/school) may contribute to this difference. These findings also highlight the necessity for more aggressive measures in the follow-up and management of children and adolescents with type 1 diabetes in Cameroon in order to reduce complications resulting from poor glycaemic control.

It is unclear whether age of the patient and duration of diabetes impact on glucose control. Studies performed in the UK and France indicate that older age and longer duration of diabetes is associated with poor glycaemic control [21]. This contrasts with a study by Elbargi et al. [34] among insulin dependent type 1 diabetics in Sudan describing a higher incidence of poor glycaemic control among younger type 1 diabetics. However, age was not associated with glycaemic control in studies from Australia, New Zealand, Egypt and the US [5, 12, 35]. This is in line with our study, where we also did not observe a significant association between age or duration of diabetes and glycaemic control.

Socioeconomic status (SES) has been recognized as an important determinant of type 1 diabetes outcome [13]. In the present study, it was noticed that children from low/middle SES displayed poor glycaemic control. These findings are consistent with findings from other studies which have previously shown that low/middle SES was associated with poor glycaemic control among children with type 1 diabetes [14, 35, 36, 37, 38].

However, family living arrangement was not significantly associated with glycaemic control in this study, while this was an important predictor in other studies [15, 16, 39].

Our study found a significant association between the number of visits to the healthcare provider and good glycaemic control. Frequent visits to the healthcare provider allow for more frequent adjustments of insulin regimens and more educational sessions if necessary. Similarly, most but not all studies have found that more frequent visits to the diabetic clinic resulted in improved glycaemic control [5, 22, 23, 24, 27].

In addition, our study has shown a significant association between primary caregiver involvement and good glycaemic control. This was demonstrated by the significantly lower mean HbA1c (8.7%) in children whose primary caregiver was the mother compared to 12.7% in those who had a primary caregiver other than the mother. This could be explained by the fact that most often the mother is the primary caregiver in pediatric patients. Studies by Al-Odayani et al. [40] and Soheilipour et al. [41] have reported that children of mothers with more knowledge of diabetes had improved glycaemic control underscoring the importance of the mother`s level of education in the care of diabetic children.

Better treatment outcomes are being observed in children and adolescents whose parents and guardians are involved in the care of the patients. Family support and involvement of parents/guardians is an important modifiable factor and has been found to promote adherence and optimal glycaemic control in a study by Anderson et al. [42]. This study found a significant association between caregiver involvement in the diabetes-related tasks of insulin injection and BGM and good glycaemic control. This indicates the importance of the parent–child relationship in effective diabetes management. Similar findings have been reported in other studies [25, 26]. However, studies have demonstrated poor glycaemic control among children with critical parent–child relationship [43]. Thus, emphasis on optimal caregiver involvement in diabetes related tasks of children is crucial to improve health outcomes. In addition, family dynamics, developmental stages, and physiological differences relating to sexual maturity are all essential in developing and implementing an optimal diabetes regimen in adolescents.

Further, adherence to the different treatment aspects represents an important factor in determining good glycaemic control and eventually better treatment outcome. In this study, a significant association was observed between good adherence to BGM and good adherence to insulin injection and good glycaemic control. However, no such association was observed with diet. Some of the reasons given for poor adherence to insulin included forgetfulness, lack of food and the fact that it was not convenient injecting insulin in school and other public places. Also, the most common reason cited for non-adherence to BGM was laziness. Similar findings have been reported by Borus and Laffel [3]. A recent meta-analysis by Hood et al. [44] demonstrated a negative correlation between treatment adherence and HbA1c levels in type 1 diabetic children and adolescents and this was found to be independent of socio-demographic and other diabetes specific variables. Finally, Mehta et al. [19] demonstrated that greater dietary adherence was associated with lower HbA1c levels in youth with diabetes, a finding that we could not confirm in our study. The 24 h recall method of dietary analysis used in this study does not represent the habitual nutrient intake of the study population as opposed to the six item diabetes.

Self-management profile (DSMP) diet subscale used in assessing dietary adherence in Mehta et al. [19].

To our surprise we also found that being on 2 daily insulin results in better glucose control than multiple (3 or more) insulin injections. Although this is counterintuitive at first glance it may represent the fact that only children not well controlled were switched to multiple insulin injections as the “default” management is 2 daily insulin injections. Unfortunately, we did not record why children were switched to multiple injections.

The small sample size of this study might have contributed to the lack of association observed between glycaemic control and age, diabetes duration, family living arrangement and dietary adherence.

The multivariate model in this study demonstrated the importance of having a mother as the primary caregiver and minimal/moderate caregiver involvement in the task of insulin injection as significant independent predictors of good and poor glycaemic control respectively. However, it is unclear whether it is the involvement of the mother herself which is important or whether the fact that the mother is the primary caregiver represents an indicator for a setting where good glucose control can be easily achieved. “Mother as a primary caregiver” may not be the “causal factor” for better glucose control, but just an indicator. Thus, it may reflect a setting related to (but not restricted to) family dynamics, developmental stages, and physiological differences relating to sexual maturity allowing better glucose control.

Although, parental involvement in the diabetes-related tasks of children have been found to be strongly linked to optimal adherence and improved glycaemic control [42], there is need for a transition between adult supervision and self-care as the child reaches physical and sexual maturity. However, studies in children and adolescents with type 1 diabetes have reported that because of the rapid hormonal changes that antagonize the action of insulin during adolescent years and the gradual gain of independence from parents, adherence to the different treatment regimens is difficult making it also difficult to achieve and maintain target glycaemic control [3, 6]. These findings suggest that families having children with type 1 diabetes can modify certain behaviors in order to improve glycaemic control.

The prognosis of children with type 1 diabetes is still very poor in sub-Saharan Africa, with a reported life expectancy of <1 year. Due to its cross-sectional design we cannot make any statement concerning life expectancy but it is noteworthy that during the time of the survey 3 of 76 children died. It is unclear which structures have to be changed to improve the outcome of these patients but supporting education efforts of caregivers and patients as well as structures that allow stronger parental (especially maternal) involvement may improve the situation.

This study had some limitations worth mentioning. The cut-off level to define “good” glycaemic control in this study was arbitrary considering the distribution of the HbA1c values observed in the study population. In addition, given the consistently poor glycaemic control reported in most studies from sub-Saharan Africa [27, 32, 34], an HbA1c target of ≤9.0% slightly above the mean HbA1c levels attained in the developed nations was used in the study. Also most of the data used in the study was self-reported by the patient as well as parent/caregiver such as dietary, insulin and BGM adherence, recall bias could have been possible and over reporting could have resulted in falsely elevated adherence levels. The indicators for SES for patients used in this study included; parental occupation and income level, but this does not adequately reflect the socioeconomic background of those in the private sector. However, a study in urban Cameroon has shown the World Bank household amenities score as a better indicator for SES in developing economies [30]. Again, the lack of association observed between glycaemic control and age, diabetes duration and family living arrangements might be attributed to the small sample size of this study which was hospital and convenience based. In addition, the tools used in this study for the measurement of adherence are those used in the various clinics to review the children/adolescents because it was difficult finding validated tools for insulin, dietary and blood glucose monitoring adherence that could be applicable in the study setting.

Finally, the cross-sectional nature of this study cannot show elements of causality as such findings might not be a true reflection of children living with type 1 diabetes in the country.

Despite the limitations of this study, it provides for the first time data from the North West Region of Cameroon on factors associated with the outcome of children with type 1 diabetes.

## Conclusions

This study among type 1 diabetic children in Cameroon shows that the mother`s involvement in the diabetes management of their children and minimal/moderate caregiver involvement in the task of insulin injection are the most important determinants for good and poor glucose control respectively. It is currently unclear whether the direct involvement of the mother is causal or whether “mother as a primary caregiver” is just an indicator for a setting in which good diabetes treatment is possible.

## Additional file

**Additional file 1.** Parent Questionnaire.

## Abbreviations

HbA1c: glycosylated haemoglobin
BGM: blood glucose monitoring
SES: socioeconomic status
OR: odds ratio
CDiC: changing diabetes in children
UK: United Kingdom
ANOVA: analysis of variance
