# Supplementary material for: Predictors of glucose control in children and adolescents with type 1 diabetes: results of a cross-sectional study in Cameroon
Source: BMC Res Notes. 2017 Jun 12;10:207. doi: 10.1186/s13104-017-2534-8 (PMC5469010; doi:10.1186/s13104-017-2534-8)
Supplement: Supplementary file 1 — Additional file 1. Parent Questionnaire. [file 13104_2017_2534_MOESM1_ESM.doc]

**Parent Questionnaire**

**Please complete all the questions on this questionnaire which will take about 30 minutes of your time**

Patient code

1. **SOCIODEMOGRAPHIC DATA AND BACKGROUND INFORMATION**
2. Telephone contact __________________
3. Residence____________________________
4. What is your child’s date of birth (day/month/year): _________________
5. When was your child diagnosed of diabetes (day/month/year):______________________
6. What was your child`s age at diagnosis of diabetes mellitus?___________________
7. What is your child`s body weight (Kg) __________
8. What is the height of your child (m) _________
9. For how long have your child been living with diabetes? ___________
10. Primary caregiver (Please circle the right answer) Example: a. Mother b. Father c. Sister/Brother

a. Mother b. Father c. Brother/Sister d. Other – specify __________________

1. What is your child`s insulin regimen: (Please circle the right answer).
   1. Multiple daily insulin injections b. 2 daily insulin injections
2. What is the highest level of education completed by the primary caregiver of your child? (circle the right answer):
   1. No formal education b. Primary c. Secondary d. High school e. University
3. What is your family structure (select one and circle the right answer):
   1. Both parents are living together b. Single parent family c. Not living with either parent d. Orphan
4. Is there any other member of your family suffering from type 1 or type 2 diabetes? (circle the right answers)
   1. Mother b. Brother/Sister c. father d. Uncle/Aunt e. Grandparent f. None
5. At the time of diagnosis did your child have any of the following; (Circle the right answer)

- Ketones in the urine a. Yes b. No
- Altered consciousness or coma a. Yes b. No

1. How many times have your child been found to have the following in the last 6 months?

-Presence of ketones in urine_________________________

-Weakness or sweating or vomiting________________________

-Admission to hospital over the last 6 months________________

1. Does your child suffer from any of the following? (Circle the right answers).
   1. Dizziness b. Poor memory c. Lack of energy d. weakness e. Coma/Convulsion
2. How many times have your child experienced any of the above during the last 6 months ____________________________
3. Did your child suffer from any of the following when he/she was young? (Circle the right answers!)
   1. Measles b. Weight loss c. Eczema d. weakness e. Mumps f. Diarrhea h. none
4. Was your child suffering from any of the following at the time of diagnosis? (Circle the right answers!)
   1. Cold b. coma c. weakness d. stomach problems e. Weight loss f. weakness g. none

For how long did your child suffer from it before being diagnosed of diabetes? ____________________

1. **Socioeconomic status (circle the right answer)**

| **MOTHER** | **FATHER** |
| --- | --- |
| 1-Number of children | 1- Number of children |
| 2-What is your highest level of education? Please tick one!  a. None b. Primary c. Secondary d. High School  e. University | 2- What is your highest level of education? Please tick one  a)None b. Primary c. Secondary d. High School  e. University |
| 3-What is your occupation? | 3-What is your occupation? |
| 4-Can you give an estimate of your household income in a month (from all available sources) Please tick one.  a) Below 25,000frs b) 25,000frs - 50,00frs  c) 50,000frs - 100,000frs d) 100,000frs - 200,000frs  e. 200,000frs – 300,000frs f) 300,000frs – 400,000frs  g. 400,000frs and above | 4-Can you give an estimate of your household income in a month (from all available sources) Please tick one.  a) Below 25,000frs b) 25,000frs - 50,00frs  c) 50,000frs - 100,000frs d) 100,000frs - 200,000frs  e. 200,000frs – 300,000frs f) 300,000frs – 400,000frs  g. 400,000frs and above |
| 5- What is your religion?  a. None b. Catholic c. Protestant d. Muslim e. Other……………………………………. | 5- What is your religion?  a. None b. Catholic c. Protestant d. Muslim e. Other……………………………………. |

1. **DIABETES RELATED PRACTICES OF PATIENT/CAREGIVER**
2. Is there anybody at the clinic/health centre that your child can contact in case of any problems for advice? a) Yes b) No
3. Was your child given clear instructions on how to handle his/her dose of insulin or inject your insulin? a) Yes b) No
4. How many doses of insulin have your child missed in the last one week?
   - 1. None b) Between 1 – 3 times c) More than 3 times
5. How many times have your child measured his/her blood glucose at home in the last one week?

a) Every day b) More than or 3 times a week c) 1 – 2 times a week d) less than Once a week

1. What did your child eat in the last 24 hours?

During breakfast _______________________________________________________________________________________

For lunch ____________________________________________________________________________________________

During supper ________________________________________________________________________________________

Any snacks? (How many times?) _________________________________________________________________________

1. How does your child store his/her insulin? a) Refrigerator b) Pot of cold water c) Room temperature
2. In the last 24 hours, how many times did the parent/caregiver inject or supervise the insulin injection of his/her child?

a) None b) Once or twice c) All the injections

7. How involved is the parent/caregiver in the testing/measurement of his/her child`s blood glucose? Please tick!

a) No involvement

b) Reminds the child to monitor glucose or logs in the level in the diary or asks about the blood glucose level

c) Sets up the meter and does the finger prick

8. How many times did your child visit the doctor/clinic during the last 6 months?

a) None b) Between 1-3 times c) More than 3 times

9. Have your child been admitted to the hospital over the last year? a) Yes b) No. **If the answer is Yes**

-How many times? ___________________________________________________________________________________

-How many days? ____________________________________________________________________________________

-What was the reason for your child’s admission ____________________________________________________________

1. **AVAILABILITY OF INSULIN**

1. In the last 3 months, have your child ever missed getting his/her prescribed insulin from the hospital?

a) Yes b) No

2. If **Yes,** how frequently have your child missed his/her supplies?

a) Every month b) Once or twice in 3 months

3. When your child misses his/her supplies of insulin or syringes, what does he/she usually do?

a) Buy his/her own insulin b) Wait till supplies of insulin are available from the hospital
